# Parameter extraction of proton exchange membrane fuel cell based on artificial rabbits’ optimization algorithm and conducting laboratory tests

**DOI:** 10.1038/s41598-024-70886-6

**Published:** 2024-09-10

**Authors:** Faisal B. Baz, Ragab A. El Sehiemy, Ahmed S. A. Bayoumi, Amlak Abaza

**Affiliations:** 1 Mechanical Engineering Department, Faculty of Engineering, Kafrelsheikh University, Kafr El Sheikh, 33516 Egypt; 2 Electrical Engineering Department, Faculty of Engineering, Kafrelsheikh University, Kafr El Sheikh, 33516 Egypt; 3 Mathematical and Physics Engineering Department, Faculty of Engineering, Kafrelsheikh University, Kafr El Sheikh, 33516 Egypt

**Keywords:** Artificial rabbits optimisation, Proton exchange membrane fuel cell, Parameter estimation, Optimization model, Experimental tests, Energy science and technology, Engineering, Nanoscience and technology

## Abstract

Proton exchange membrane fuel cell (PEMFC) parameter extraction is an important issue in modeling and control of renewable energies. The PEMFC problem’s main objective is to estimate the optimal value of unknown parameters of the electrochemical model. The main objective function of the optimization problem is the sum of the square errors between the measured voltages and output voltages of the proposed electrochemical optimized model at various loading conditions. Natural rabbit survival strategies such as detour foraging and random hiding are influenced by Artificial rabbit optimization (ARO). Meanwhile, rabbit energy shrink is mimicked to control the smooth switching from detour foraging to random hiding. In this work, the ARO algorithm is proposed to find the parameters of PEMFC. The ARO performance is verified using experimental results obtained from conducting laboratory tests on the fuel cell test system (SCRIBNER 850e, LLC). The simulation results are assessed with four competitive algorithms: Grey Wolf Optimization Algorithm, Particle Swarm Optimizer, Salp Swarm Algorithm, and Sine Cosine Algorithm. The comparison aims to prove the superior performance of the proposed ARO compared with the other well-known competitive algorithms.

## Introduction

Life requires energy. Energy is one of humanity’s most pressing concerns. Increasing energy demand, regional constraints, and the serious environmental consequences of traditional energy sources highlight the urgent need for new, clean, and sustainable energy solar photovoltaic (PV) cells /modules with estimated parameters of the electrical models^1–4^, wind energy^5,6^, hydro, and some electrochemical. The need to examine different sources of energy is not the only way to support the energy requirements; power management^7,8^, new materials that minimize losses^9–12^, and energy harvesting^13^ have become more crucial in recent years.

Hydrogen has the advantage of being produced in various ways, including using renewable energy sources. As an energy carrier, hydrogen can also be used for energy storage and is easily converted to heat and electric energy via fuel cells. Hydrogen might also be employed in mobility because this technology is eco-friendly.

One of these applications is the fuel cell, a type of electrochemical device composed of an anode connected via an electrolyte to a cathode that produces electrical power when fuel-fed^14^. Fuel cells are classified according to the fuel and electrolyte used.

Proton exchange membrane fuel cells (PEMFCs) are among the most prominent of these electrochemical energy conversion technologies^15^. The main advantages of such technology in power systems applications are high startup reliability, low carbon emissions, low startup costs, quiet operation, and quick response to demand changes^16^. For these factors, PEMFCs are currently used to power hydrogen-powered cars in the international marketplace, where they account for roughly 90% of fuel cell advancements^17^. As a result, in addition to other renewable energy sources, hydrogen might fuel PEMFC reactors operating at partial loading conditions, yielding a significant swift load cover^18^. There has been a rise in studies into fuel cell calculation of parameters in recent years^19^. Because PEMFC is an integrated multivariable powerfully combined scheme, it might, for example, be used in the combined difficult problem. Many techniques for obtaining precise values for these parameters have been proposed.

Meta-heuristic optimization approaches are, in particular, significant, hopeful, and strong to acquire accurate PEMFC model parameters because of the simplicity with which they can be implemented, reproducibility, durability, and simplicity. To produce more effective heuristic solutions to optimization challenges, metaheuristic methods can often be motivated by real-world occurrences, such as emulating physical principles or phenomena in biology. There are two types of metaheuristic methods: swarm-based strategies and evolutionary steps.

These algorithms are stronger and more broadly applicable to global optimization techniques than the traditional optimization technique in solving engineering problems as Enhanced Real Coded Genetic Algorithm^8^ has been used to optimum Distributed Generation (DG) positioning in a Radial Distribution System, The filter design in electrical circuit was optimized by an enhanced Tree-Seed Algorithm in^20^.

The Solid Oxide Fuel Cell (SOFC) parameters were optimized for steady state and transient simulations using the interior search algorithm^21^, a comparison of parametric estimation methods for a PEMFC using metaheuristic algorithms^22^ has been performed by Tabbi Wilberforce et al., Optimal parameter estimation strategy of Proton Exchange Membrane (PEM) has been performed fuel cell using gradient-based optimizer^23^, the hybrid grey wolf optimization method^24^ has been utilized to estimate the PEMFC parameters, for maximum power point tracking (MPPT)^25^ an innovative approach based on a recently developed equilibrium optimizer for improving the efficacy of a PEMFC system using optimized fuzzy logic, bald eagle search optimizer^26^ used for parameter estimation of SOFCs, coyote optimization algorithm^27^ used for Optimal parameter estimation of SOFC, a modified crow search optimizer^7^ in order to solve the non-linear optimal power flow (OPF) problem with emissions, marine predators optimizer^1^ for effective PV parameter estimation taking into account low radiation in addition to normal operating conditions , closed loop particle swarm and elephant herd^28^ used for comparing modified two-diode to improved three-diode patterns of multi-crystal solar cells.

As demonstrated by the preceding concise survey in a similar context, there continues to be advancement within the domain of optimization methods, and no single algorithm can solve all optimization problems, according to the no-free-launch theorem. This later encouraged numerous researchers to put their skills and obtain enhanced attributes to meet their challenges in various scientific and technical fields. Natural rabbit survival strategies such as detour foraging and random hiding are inspired by Artificial rabbit optimization (ARO)^29^. To discuss the motivation of the ARO, it is the endurance approaches of rabbits in nature, comprising diversion hunting and random beating. The diversion hunting approach imposes a rabbit to eat the grass near other rabbits’ nests, which can prevent its nest from being discovered by predators. The random hiding approach allows a rabbit to randomly select one hole from its own holes for hiding, which can decline the option of being captured by its enemies. Besides, the energy shrink of rabbits will result in the transition from the detour foraging strategy to the random hiding strategy^29^.

Several applications are for the ARO in different fields as extracting the Parameters of PEMFC^30^, quantum ARO was developed for energy management in microgrid taking into account demand response^31^, parameter estimation of PVs cells/modules^32,33^, feature selection in medical applications^34^, developed a dynamic models for multi-Layer Perovskite Solar Cell^35^, for designing the proportional integral and derivative (PID) controller for enhancing the operation of load frequency in Multi-area grids^36,37^, and for enhancing the operation of distribution systems with compensation devices^38^

Meanwhile, the rabbit energy shrink is emulated to control the smooth switching from a detour foraging state to a random hiding state. The main impacts of this effort are highlighted in the next statements:

- ARO is proposed as a profitable candidate technique for optimizing PEMFC model parameters.
- The proposed algorithm is validated using experimental results obtained from laboratory tests of the fuel cell test system (SCRIBNER 850e, LLC).
- The superiority of ARO is demonstrated by contrasting it with various solution methodologies found in the literature.

The remainder of this paper’s text has been divided as follows: Section "PEM Fuel Cell Model" explains a theoretical PEMFC model. Section "Parameter Estimation of PEMFC and ARO" describes the ARO procedures in detail and the adapted formulations of problems regarding the objective function and associated restrictions. Section “Results and discussions” discusses simulation results. Finally, Section "Assessment Study" summarizes the final remarks and prospects for expanding this current effort.

## PEM fuel cell model

### Operation of PEMFC

The PEMFC comprises three basic components: an anode, a proton exchange membrane, and a cathode. The ionization of hydrogen gas produces protons and electrons. The anode site’s reaction is given below:

1 $$2{H}_{2} \rightleftarrows 4{H}^{+}+4{e}^{-}$$

At the cathode site, Oxygen reacts with circuit electrons and electrolyte protons. (Proton exchange membrane) to form water.

2 $${ 4{H}^{+}+4{e}^{-}+{O}_{2} \rightleftarrows 2H}_{2}O$$

The overall reaction of the cell can be represented as:

3 $$2{H}_{2}+{O}_{2}\rightleftarrows 2{H}_{2}O$$

### The PEMFC output voltage

The output voltage of a single PEMFC, ${V}_{fc}$, is the result of thermodynamic potential, ${E}_{Nernst}$, and potential losses during the conversion process^39,40^.

4 $${V}_{fc}={E}_{Nernst}-(All \;the \;fuel \;cell \;losses )$$

5 $${E}_{Nernst}=1.229-\left(8.5\times {10}^{-3}\right)\left(T-298.15\right)+\left(4.308\times {10}^{-5}\right)T\text{ln}{(P}_{{H}_{2}}^{*}\times \sqrt{{P}_{{O}_{2}}^{*}})$$

where *T* is the temperature in Kelvin (${P}_{{O}_{2}}^{*}$) is oxygen partial pressure and (${P}_{{H}_{2}}^{*}$) is hydrogen partial pressure, which can be calculated by:

6 $${P}_{{H}_{2}}^{*}=\frac{{RH}_{a}.{P}_{{H}_{2}O}}{2}[\left(1/\frac{{RH}_{a}.{P}_{{H}_{2}O}}{{P}_{a}}{e}^{(1.635\left(\frac{I}{A}\right) /{T}^{1.334})}\right)-1]$$

7 $${P}_{{O}_{2}}^{*}={RH}_{c}.{P}_{{H}_{2}O}[\left(1/\frac{{RH}_{c}.{P}_{{H}_{2}O}}{{P}_{c}}{e}^{(4.192\left(\frac{I}{A}\right) /{T}^{1.334})}\right)-1]$$

where $RH_{a} { }\;\;{\text{and}}\;\;RH_{c}$ are relative humidity of vapor at the anode and cathode, respectively, $P_{{H_{2} O}}$ is the water vapour saturation pressure in atm, given by^41^:

8 $$\log_{10} (P_{{(H_{2} O)}} ) = 1.44 \times 10^{ - 7} (T - 273.15)^{3} - 2.18 + 2.95 \times 10^{ - 2} \left( {{\text{T}} - 273.15} \right) - 9.18 \times 10^{ - 5} \left( {{\text{T}} - { }273.15} \right)^{2}$$

**Fig. 1:**
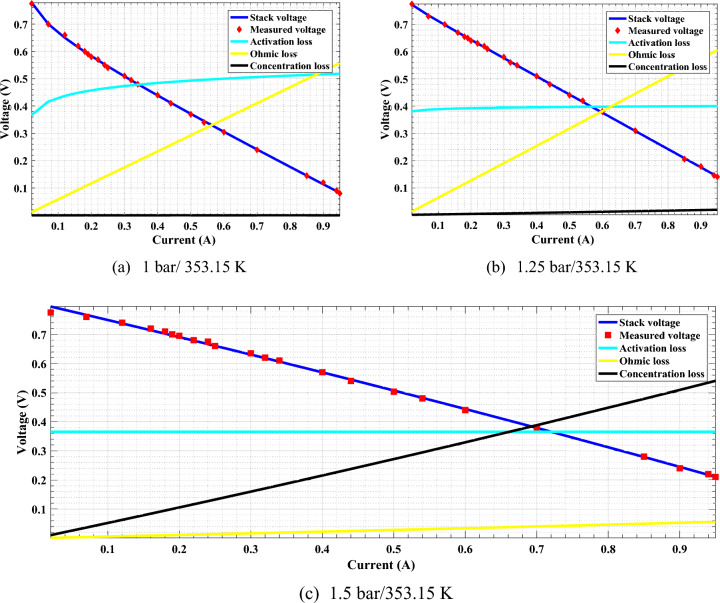
Polarization (V-I) curve of PEMFC at (**a)** 1.0 bar/ 353.15 K, (**b)** 1.25 bar/353.15 K, and **c** 1. 5 bar/353.15 K.

Three types of losses are associated with PEMFC: activation, ohmic, and concentration losses, which cause the fuel cell’s output voltage to vary with load current^42^. The voltage losses of PEMFCs at various current levels are depicted in Fig. 1.

The first and largest type of loss is activation cell loss, which starts early in the reaction. This is due to the sluggishness of the reaction caused by electronic barriers that had to be surpassed^43^. It can be described as the amount of voltage lost to allow ions to pass from one electrode to another and can be calculated as^44–46^:

9 $${V}_{a}=-\left[{\upxi }_{1}+{\upxi }_{2}T+{\upxi }_{3}T\left(\text{ln}\left({C}_{{O}_{2}}^{*}\right)\right)+{\upxi }_{4}T(\text{ln}\left(I\right))\right]$$

where the factors ${\upxi }_{1}-{\upxi }_{4}$ are activation loss voltage coefficients as a function of actual temperature, T (K), oxygen concentration, ${C}_{{O}_{2}}^{*}$, and load current, I.

The actual temperature and oxygen partial pressure ${P}_{{O}_{2}}^{*}$ affect the ${C}_{{O}_{2}}^{*}$ value.

10 $${C}_{{O}_{2}=}^{*}{[P}_{{O}_{2}}^{*}/(5.08\times {10}^{6})]{e}^{(498.15/T)}$$

The ohmic voltage loss is the second type of loss. It results from proton flow resistance in solid polymer membranes and electron transfer resistance through cell electrodes.

11 $${V}_{ohmic}={V}_{ohmic}^{electronic}+{V}_{ohmic}^{proton}= I{R}_{c} +{ IR}_{m}$$

where ${R}_{c}$ represents electronic resistance value, which is unknown and thought to be roughly constant throughout the cell’s operating conditions, ${R}_{m}$, represents the proton membrane resistance, which is determined by the membrane’s specific resistivity, ${\rho }_{m} (ohm.cm)$, area A (cm^2^), and thickness $l (cm)$. The thickness considered for the Nafion membrane could be [115:5 mil (127 m)]^41,44,45^.

12 $${R}_{m}={\rho }_{m} l /A$$

The following empirical expression can be used in the calculation of ${\rho }_{m}$ value^41^:

13 $${\rho }_{m}=\frac{181.6[1+0.03\left(\frac{I}{A}\right)+0.062{\left(\frac{T}{303}\right)}^{2}.{\left(\frac{I}{A}\right)}^{2.5}}{[ \lambda -0.634-3\left(\frac{I}{A}\right){e}^{\left(4.18\left[T-303\right]/T\right)}]}$$

The adjustable parameter $\lambda$, demonstrates the membrane’s effective water content. It could range from 14 in ideal, 100% relative humidity levels to 22 and 23 in supersaturated levels^41^. The concentration loss,${V}_{conc.}$, is the third voltage loss of a PEMFC. It occurs at high limiting currents (${I}_{max}$), where the concentration decreases due to the opposition of getting enough reactants to the electrode surface^44–46^.

14 $${V}_{conc.}=-b\times \text{ln}(\left({J}_{max}-J\right)/{J}_{max}$$

where $b$ denotes the unknown coefficient.

Equation (15) determines the output voltage of PEMFC. If ${n}_{cells}$ are series cells that connected to achieve the needed voltage, the resulting stack voltage is the sum of all cells’ output, as shown below^45,47^:

15 $${V}_{fc}={E}_{Nernst}-\left({V}_{a}+{V}_{ohmic}+{V}_{conc.}\right)$$

## Parameter estimation of PEMFC and ARO

### The parameters of PEMFC

The primary goal of the PEMFC issues is to estimate the optimal value of unknown parameters of the electrochemical model. The optimized parameters are identified so that the summation of square error (SSE) between the model simulation voltage ‘${V}_{fc}$’ and the measured voltage ‘${V}_{meas}$’ is minimized. The simulated model optimizes seven parameters ($\small {\upxi }_{1}, {\upxi }_{2},{\upxi }_{3}, {\upxi }_{4}, {R}_{c}, \lambda, and, b)$ described in Eqs. (9)–(14). ^26,48^

16 $$SSE=\sum_{i=1}^{n}{[{V}_{meas}\left(i\right)-{V}_{fc}\left(i\right)]}^{2}$$

where *n* denotes the number of measured voltages at different load currents. The optimization problem’s objective function aims to o achieve the minimum value of SSE, as follows^26^:

17 $$OF=\text{min}(SSE)$$

The unknown parameters are optimized so that their values are within the allowed minimum and maximum limits, as follows: -

18 $$\begin{array}{c}{\upxi }_{\text{i}}^{\text{min}}{\le\upxi }_{\text{i}}\le {\upxi }_{\text{i}}^{\text{max}} for i=\text{1,2},3 \;and \;4\\ {\text{R}}_{\text{c}}^{\text{min}}{\le \text{R}}_{\text{c}}\le {\text{R}}_{\text{c}}^{\text{max}}\\ \begin{array}{c}{\lambda }^{min}\le \lambda \le {\lambda }^{max}\\ {b}^{min}\le b\le {b}^{max}\end{array}\end{array}$$

### Artificial rabbits optimization (ARO):

Natural rabbit tactics for survival inspired Artificial rabbit optimization (ARO), such as detour foraging and random hiding. Meanwhile, rabbit energy shrink is mimicked to control the smooth switching from the detour foraging to the random hiding strategy. ARO was created to solve global optimization problems with a single objective^29^.

#### Detour foraging (exploration)

As stated, rabbits look far away as foraging and ignore what is readily available. They only eat grass randomly in other areas rather than in their own; we call this foraging behavior “detour foraging.” In ARO, suppose that every rabbit among the swarm possesses its own area, including grass and burrows, and that the rabbits forage randomly at each other’s positions. So, ARO’s detour foraging behavior indicates that every search individual tends to update its location in relation to another search individual chosen randomly from the swarm and adds a perturbation.

The mathematical model shown below of the rabbit foraging detour is proposed:

19 $$\begin{gathered} p_{i} (z + 1) = x_{j} (z) + R.(x_{i} (z) - x_{j} (z)) + round(0.5.(0.05 + r_{1} )).n_{1} ,\quad \hfill \\ i,j = 1,...,n\quad and\quad i \ne j \hfill \\ \end{gathered}$$

20 $$R = L.c$$

21 $$L = (e - e^{{(\frac{z - 1}{Z})^{2} }} ).\sin (2\pi r_{2} )$$

22 $$c(k) = \left\{ {\begin{array}{*{20}c} {1\quad if\;k = = g(l)} \\ {0\quad \quad \quad \quad else} \\ \end{array} } \right.\;\quad k = 1,...,d\;and\;l = 1,...,\left[ {r_{3} .d} \right]$$

23 $$g = randperm(d)$$

24 $$n_{1} \sim \;N(0,1)$$

where z_i_(t + 1) is i_th_ rabbit’s candidate location at time z + 1, xi(t) is i_th_ rabbit’s location at time z, n is the size of a rabbit population, d is the problem dimension, and Z is the maximum number of iterations, ⌈⋅⌉ is ceiling function, means rounding to the closest integer, $randperm$ brings back a permutation of the integers at random from 1 to d, r_1_, r_2_, and r_3_ are three random numbers in (0,1), L stands for the speed at which the detour foraging is performed, and n1 is in accordance with the standard normal distribution. c is a mapping vector that can assist the algorithm in randomly selecting a search individual’s random number to change the foraging behavior. R is a running operator used to simulate rabbit running behavior.

According to Eq. (19), search individuals conduct a random search for food based on their location. This behavior enables a rabbit to travel long distances from its own area to areas of other rabbits. This unique foraging behavior of rabbits that visit other people’s nests rather than their own contributes significantly to exploration and ensures the ARO algorithm’s global search capability.

#### Random hiding (exploitation)

The i_th_ rabbit’s j_th_ burrow is generated by:

25 $$b_{i,j} = x_{i} (z) + H.g.x_{i} (z),\;i = 1,...,n\;and\;j = 1,...,d$$

26 $$H = \frac{Z - z + 1}{Z}.r_{4}$$

27 $$n_{2} \sim \;N(0,1)$$

28 $$g(k) = \left\{ {\begin{array}{*{20}c} 1 & {if\;k = = j} \\ 0 & {else} \\ \end{array} } \right.\quad k = 1,...,d$$

Equation (15) generates d holes in the location of a rabbit in random with each dimension. H stands for hiding parameter, which is reduced linearly from 1 to 1/T with a random alarm throughout iterations. Initially, these holes are made in a larger neighbourhood of a rabbit, according to this parameter. If the number of iterations rises, then the size of this neighbourhood declines. Equations (29)- (32) are planned to mathematically model the random hiding strategy as:

29 $$p_{i} (z + 1) = x_{i} (z) + R.(r_{4} .b_{i,r} (z) - x_{i} (z)),\quad i = 1,...,n\quad$$

30 $$g_{r} (k) = \left\{ {\begin{array}{*{20}c} {1\;\;\;\quad if\;k = = [r_{5} .d]} \\ {0\quad \quad \quad \quad else} \\ \end{array} } \right.\;\quad k = 1,...,d\;$$

31 $$b_{i,r} (z) = x_{i} (z) + H.g_{r} .x_{i} (z)$$

where b_i, r_ is a burrow chosen randomly to hide from its d burrows, and *r*_4_ and *r*_5_ are both numbers chosen randomly in (0,1). Based on Eq. (29), the *i*_th_ search individual modifies its location in relation to the randomly chosen burrow from its d burrows.

When one of random hiding or detour foraging is done, the i^th^ rabbit position is updated as:

32 $$x_{i} (z + 1) = \left\{ {\begin{array}{*{20}c} {x_{i} (z)\;\;\;\quad f(x_{i} (z)) \le f(p_{i} (z + 1))} \\ {p_{i} (z + 1)\quad \quad f(x_{i} (z)) \ge f(p_{i} (z + 1))} \\ \end{array} } \right.\;\quad$$

According to this equation, if the fitness of the ith rabbit’s candidate position is higher than that of the present position, the rabbit will leave its present location and remain at the candidate one generated by Eq. (19) or Eq. (29).

#### Energy reduction (change from exploration to exploitation)

In ARO, rabbits frequently employ detour foraging in the early iterations while randomly hiding in the later iterations. This search mechanism is powered by a rabbit’s energy, which gradually depletes over time. As a result, an energy factor is created to model the transition from exploration to exploitation. ARO energy factor is described as:

33 $$A(z) = 4\left( {1 - \frac{z}{Z}} \right)\ln \left( \frac{1}{r} \right)$$

where $r$ is a random number between (0,1).

**Fig. 2:**
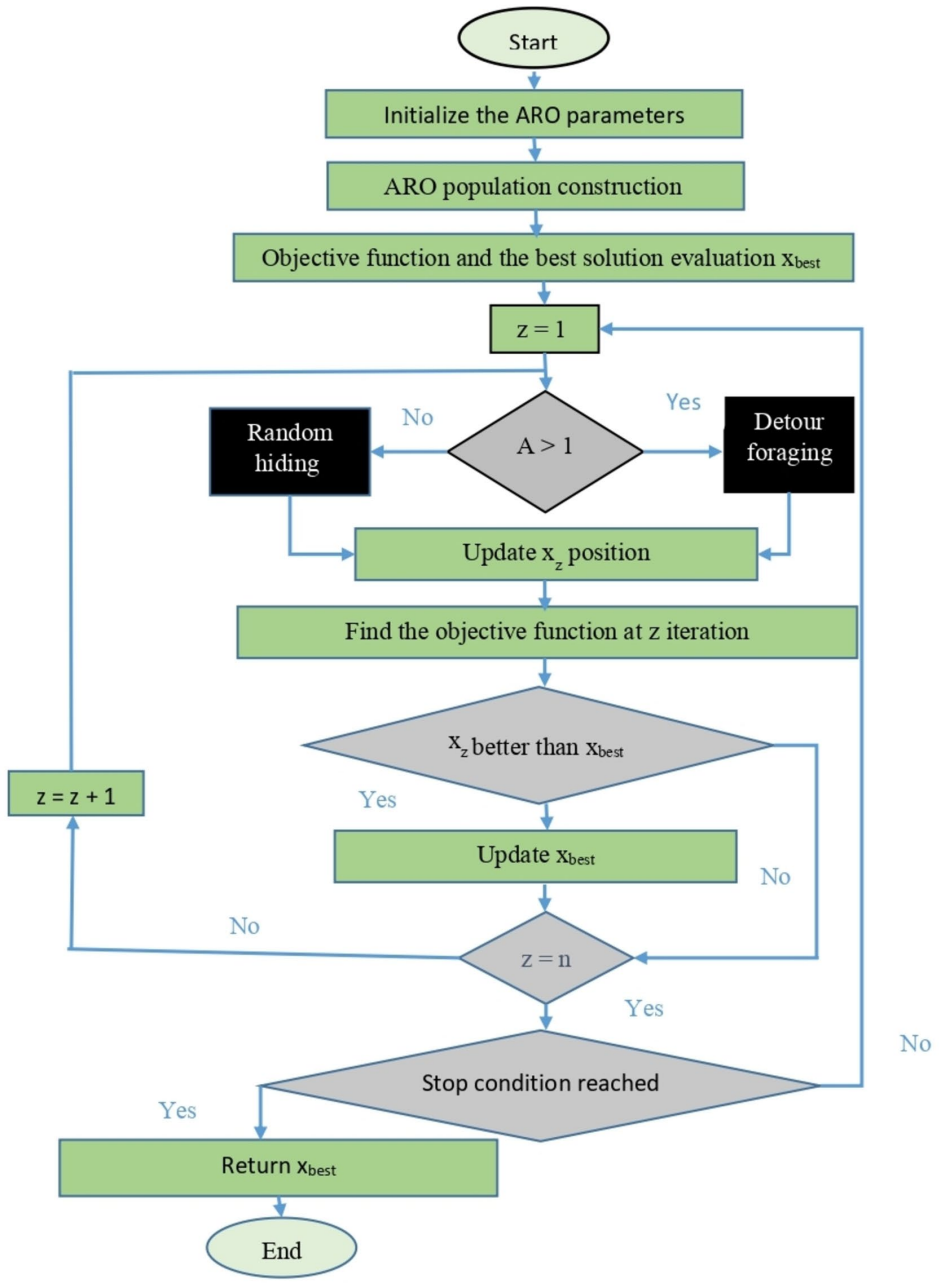
The ARO flowchart.

The large energy factor value shows that the rabbit has enough energy and physical stamina for detour foraging. On the other hand, a low energy factor indicates that the rabbit is less energetic and thus needs random hiding. ARO flowchart is shown in Fig. 2.

## Results and discussions

**Fig. 3:**
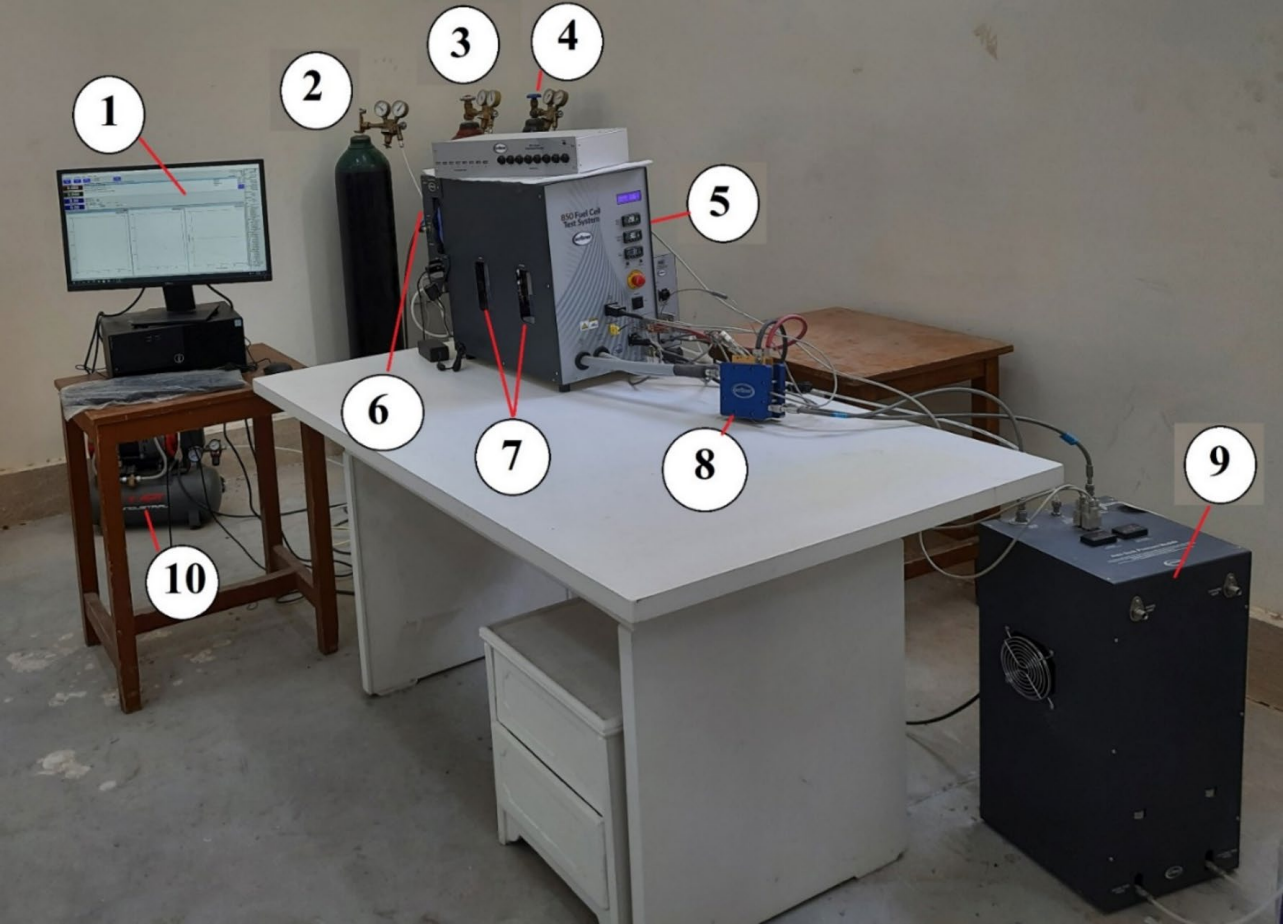
A real image of the fuel cell test system.

The proposed algorithm is verified using experimental results obtained from conducting laboratory tests on a fuel cell test system (SCRIBNER 850e, LLC). Figure 3 shows an image of the fuel cell test system and its components. These components are denoted as: (1) Computer for control and monitoring, (2) Nitrogen cylinder, (3) Hydrogen cylinder, (4) Oxygen cylinder, (5) Fuel cell test station, (6) Multi-gas selector, (7) Anode and cathode humidifier, (8) Fuel cell, (9) Back-pressure unit, and (10) Air compressor. The fuel cell test system consists of many systems. Firstly, the reactants supply system involves a hydrogen, Oxygen, and air compressor. In addition, a nitrogen cylinder is used for the purging process.

Secondly, the fuel cell unit consists of a polymer electrolyte membrane (NAFION™ 117) and anode and cathode catalyst layers with a loading of 0.5 gm of platinum / cm^2^ of the catalyst layer and 60% of platinum on Vulcan (Carbon). Gas diffusion layers (CT GDS090S Carbon Fiber Paper) are used in both the anode and cathode sides to distribute uniform reactants and prevent flooding. Gold-plated copper current collectors are used on both sides to transfer the current to the external circuit. Conventional serpentine flow fields of graphite are used for reactant distribution through the cell.

**Fig. 4:**
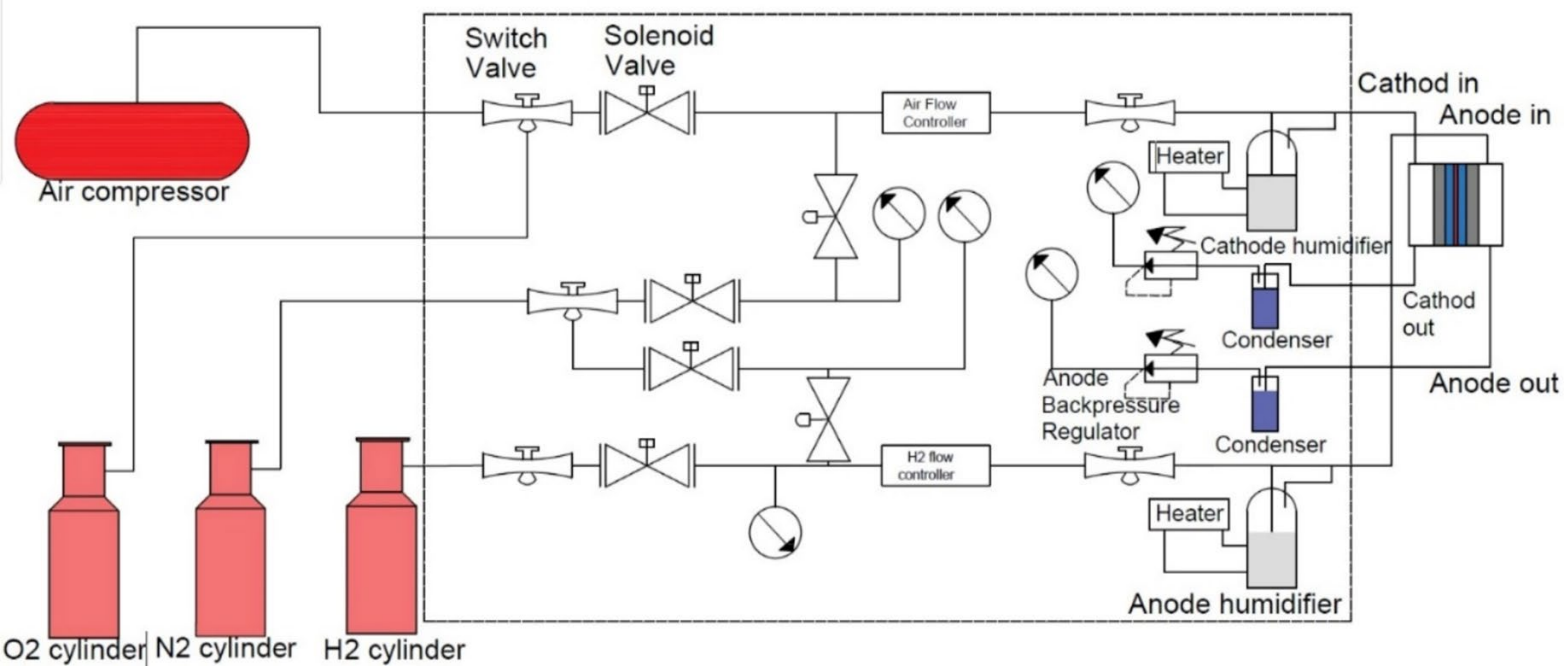
A schematic diagram of the fuel cell test system.

Thirdly, a SCRIBNER 850e fuel cell test system controls and monitors the fuel cell operation. It consists of an Automated Multi-Gas Selector to switch between reactants, an Automated Humidifier system to control the reactant’s relative humidity, an Automated Back Pressure unit to control the operating pressure and heaters to control the cell temperature. This brilliant system provides a wide voltage and current range and can be operated in a wide temperature range and reach 120 °C. The system specification and operating parameters are illustrated in Table 1. Furthermore, the cell performance could be monitored and recorded using the fuel cell test station. Figure 4 shows a schematic diagram of the fuel cell test system. Table 2 lists the tested cell’s parameters, technical specifications, and operating circumstances. The cell has been tested at three different operating pressures (1.0, 1.25, and 1.5 bar) and a temperature of 353.15 K. For all experiments, the fuel cell runs according to operating conditions shown in Table 1 until its operation reaches a steady state case for around thirty minutes. After that, the pressure is adjusted to the required value, and the cell works to get the performance curve.

**Table 1:** PEMFC technical data and operating conditions.

Technical data		Operating conditions	
Number of cells in series	1	*P*_*a*_ (bar)	*1,1.25,1.5*
Active area of one cell (cm^2^)	5	*P*_*c*_ (bar)	*1,1.25,1.5*
Nafion 117 thickness (µm)	183	Cell temperature *T* (K)	353.15
Maximum current density J_max_ (A cm^-2^)	1.1	Relative humidity in anode *RH*_*a*_	1
Rated power (W)	5	Relative humidity in cathode *RH*_*c*_	1
Reactants	H_2_ and Air

**Table 2:** Parameters, technical, and operating circumstances of the tested cell.

Quantity	Unit	Value	Quantity	Unit	Value
Number of cells in series	–	1	GDL porosity	%	68
anode relative humidity	%	100	GDL permeability	m^2^	1 × 10^–12^
Anode stoichiometric ratio (SR)	–	1.25	GDL thickness	m	90 × 10^–6^
Anode inlet temperature	K	353.15	CL porosity	–	0.112
Anode dew point	K	353.15	CL permeability	m^2^	2 × 10^–13^
Anode Outlet pressure	bar	1/1.25/1.5	CL thickness	m	215 × 10^–6^
Cathode relative humidity	%	100	Dry membrane density	Kg/m^3^	1980
Cathode SR	–	2.0	Membrane thickness	m	183 × 10^–6^
Cathode inlet temperature	K	353.15	Membrane water uptake (353.15 K)	%	34
Cathode dew point	K	353.15	Channel height	m	1 × 10^–3^
Cathode Outlet pressure	bar	1/1.25/1.5	Channel width	m	1 × 10^–4^
Operating temperature	K	353.15	Rib width	m	1 × 10^–4^
Open circuit voltage	V	0.96	Fuel cell area	m^2^	5 × 10^–6^

The control variables (${\upxi }_{1},{\upxi }_{2},{\upxi }_{3}, {\upxi }_{4}, {R}_{c}, \lambda, and, b)$ have been estimated optimally based on the ARO algorithm using Eqs. (19–33) (5–17). An accurate PEMFC model can be established when the optimization problem is solved accurately to extract the model parameters. In our optimization problem, the objective function is the summation of the square error (SSE) between the output voltages of the proposed electrochemical optimized model with respect to the measured voltages at various loading conditions. The simulation results are assessed with four competitive algorithms: Grey Wolf Optimization Algorithm (GWO), Particle Swarm Optimizer (PSO), Salp Swarm Algorithm (SSA), and Sine Cosine Algorithm SCA. For a fair comparison, the population size of all algorithms and the number of iterations is taken the same. The population size is equal to 150 and the number of iterations is set at 500 for all algorithms. The comparison aims to prove the superior performance of the proposed ARO compared with the other well-known competitive algorithms. The control parameters of the competitive algorithms are described in Table 3.

**Table 3:** Settings of the control parameters of each optimizer.

Algorithm	Control Parameter	Value
ARO	No control parameters	–
GWO	Convergence control parameter	a: linear reduction factor from 0 to 2
PSO	Cognitive and social constants Inertia weight	C_1_ = 2, C_2_ From 0.9 to 0.4
SCA	Constant to adapt function range	a = 2
SSA	No control parameters	–

Tables 4, 5, 6 summarize the optimal parameters of the PEMFC based on the electrochemical model estimated by four competitive optimization algorithms besides the lower and upper limits of the PEMFC control variables. The simulation results are reported at three operating conditions (1.0, 1.25, and 1.5 bar at 353.15 K). The extracted parameters from Tables 4, 5, and 6 agree with the measured ones at lower SSE values. It is found that the ARO algorithm outperforms the others as it has the lower value of SSE (2.6918E−04, 1.6077E−04, and 7.9759E−04) at operating conditions of 1.0, 1.25, and 1.5 bar at 353.15 K; respectively. The simulation results in Tables 4, 5, and 6 assure the superiority of ARO compared to the competitive optimizers.

**Table 4:** Optimal parameters of PEMFC extracted by different optimizers at 1.0 bar/353.15 K.

parameter	Boundary		Optimal parameters				
	Lower	Upper	ARO	GWO	PSO	SCA	SSA
${\upxi }_{1}$	−0.9	−0.55	-0.695862	−0.55	−0.55	−0.55	−0.5713761
${\upxi }_{2}$	1E−4	1E−1	1.7770E−03	9.2247E−05	8.9609E−05	1.1611E−04	1.6489E−04
${\upxi }_{3}$	3E−7	9.76E−5	8.7334E−05	5.5323E−07	3.6000E−07	2.4066E−06	1.4046E−06
${\upxi }_{4}$	−26E−7	−9.5E−11	−1.1066E−04	−1.1066E−04	−1.1065E−04	−1.1191E−04	−1.1086E−04
$\lambda$	13	23	22.9997464	23	23	23	22.9399761
${\text{R}}_{\text{c}}$	1E−6	1.5	5.5504E−01	5.5491E−01	5.5507E−01	5.5339E−01	5.5472E−01
$b$	1.4E−4	0.1	1.0389E−04	1.0000E−04	1.0000E−04	1.3600E−04	1.0000E−04
SSE			2.6918E−04	2.6922E−04	2.6918E−04	2.7033E−04	2.6923E−04

**Table 5:** Optimal parameters of PEMFC extracted by different optimizers at 1.25 bar/353.15 K.

parameter	Boundary		Optimal parameters				
	Lower	Upper	ARO	GWO	PSO	SCA	SSA
${\upxi }_{1}$	−0.9	−0.55	−0.5629776	−0.5864007	−0.9	−0.9	−0.8754715
${\upxi }_{2}$	1E−4	1E−1	6.6608E−04	6.0795E−04	2.8081E−03	1.4240E−03	1.4361E−03
${\upxi }_{3}$	3E−7	9.76E−5	1.4377E−05	5.7078E−06	9.7600E−05	3.6000E−07	6.0167E−06
${\upxi }_{4}$	−26E−7	−9.5E−11	−1.4177E−05	−1.4625E−05	−1.4123E−05	−1.3017E−05	−1.3402E−05
$\lambda$	13	23	13.0018171	13.1756029	13	17.72359778	22.3316842
${\text{R}}_{\text{c}}$	1E−6	1.5	0.57890299	0.58906813	0.57891533	0.616265283	0.6259662
$b$	1.4E−4	0.1	0.09995103	0.0504913	0.1	2.47436E−05	0.00292857
SSE			1.6077E−04	1.6359E−04	1.6077E−04	1.7481E−04	1.7464E−04

**Table 6:** Optimal parameters of PEMFC extracted by different optimizers at 1.5 bar/353.15 K.

parameter	Boundary		Optimal parameters				
	Lower	Upper	ARO	GWO	PSO	SCA	SSA
${\upxi }_{1}$	−0.9	−0.6	−0.677947	−0.600867	−0.68153	−0.9	−0.600023
${\upxi }_{2}$	1E−4	1E−1	0.0009945	0.0009015	0.0009	0.0017726	0.0009
${\upxi }_{3}$	3E−7	9.76E−5	7.84E−06	1.68E−05	3.60E−07	1.84E−05	1.69E−05
${\upxi }_{4}$	−26E−7	−9.5E−11	−1.31E−10	−1.35E−10	−9.54E−11	−2.63E−10	−4.27E−10
$\lambda$	13	23	13.000062	13	13	13	13
${\text{R}}_{\text{c}}$	1E−6	1.5	1.42E−06	0.0001074	1.00E−06	1.00E−06	1.00E−06
$b$	1.4E−4	4	2.8498441	2.8496331	2.8498928	2.8565369	2.8498769
SSE			7.9759E−04	7.9766E−04	7.9759E−04	8.0087E−04	7.9760E−04

**Fig. 5:**
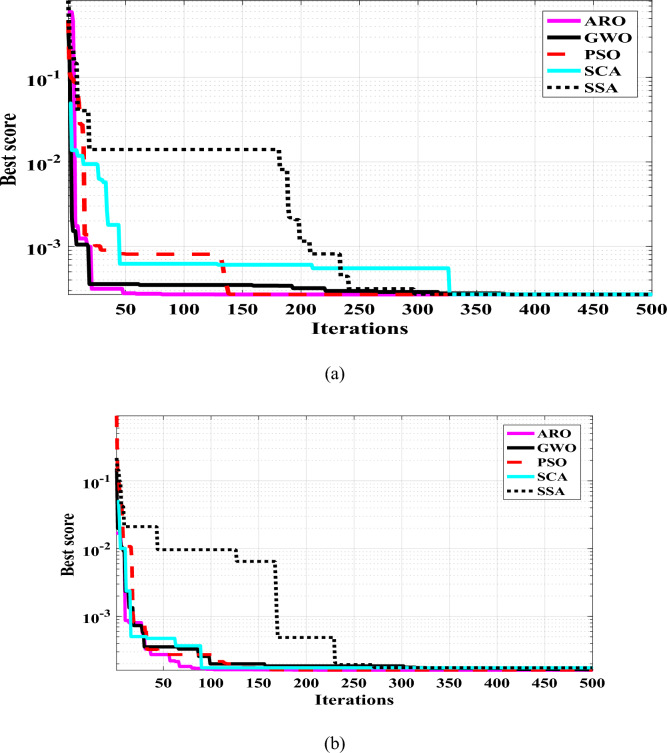
Convergence rate of 5- W PEMFC at (**a)** 1.0 bar/ 353.15 K, (**b)** 1.25 bar/353.15 K.

The convergence rate of the proposed ARO is illustrated in Fig. 5, compared to other competing algorithms. It is clear that the proposed ARO has a fast convergence rate and the best objective function (lower SSE) compared to the other optimizers. To better evaluate the proposed ARO’s performance, the competitive optimizers are executed in 30 Runs. Statistical indices for the algorithms used in our study are shown in Table 7, 8, 9. From Tables 7, 8, and 9, the outperformance of the proposed ARO algorithm is proved through all operating conditions. The proposed ARO algorithm performs better than the four optimizers used in this study. From Table 7, for PEMFC at 1.0 bar/353.15 K, the SSE values have fluctuated between 2.6918E−04 and 2.7033E−04. The minimum SSE of 2.6918E−04 is given by the ARO and PSO algorithms flowed by 2.6922E−04 applying the GWO, 2.6923E−04 via SSA, and 2.7033E−04 with the SCA algorithm. Moreover, the lower values of average SSE, variance, median, and STD are found via the ARO algorithm. Through the other two operating conditions (1.25 bar/353.15 K and 1.5 bar/353.15 K), the statistical indices in Tables 7 and 8 show the best accuracy of ARO among the other algorithms. The details of 30 Runs for the PEMFC model are reported in Tables 10, 11, 12.

**Table 7:** Statistical indices of applied optimizers at 1.0 bar/353.15 K (30 times).

Metric	ARO	GWO	PSO	SCA	SSA
Best	2.6918E−04	2.6922E−04	2.6918E−04	2.7033E−04	2.6923E−04
Worst	2.6925E−04	2.8063E−04	9.6598E−02	7.2226E−04	1.4383E−03
Average	2.6919E−04	2.7213E−04	3.8188E−03	3.6724E−04	4.2434E−04
Variance	3.1427E−16	1.2518E−11	3.0712E−04	8.1237E−09	5.6427E−08
Median	2.6919E−04	2.7055E−04	7.4808E−04	3.3680E−04	3.5941E−04
STD	1.7728E−08	3.5381E−06	1.7525E−02	9.0132E−05	2.3754E−04

**Table 8:** Statistical indices of applied optimizers at 1.25 bar/353.15 K (30 times).

Metric	ARO	GWO	PSO	SCA	SSA
Best	1.6077E−04	1.6359E−04	1.6077E−04	1.7481E−04	1.7464E−04
Worst	1.6403E−04	2.3694E−04	9.6875E−03	3.1797E−04	6.7729E−03
Average	1.6148E−04	1.7977E−04	2.3547E−03	2.3743E−04	1.7409E−03
Variance	5.6496E−13	4.8194E−10	1.6113E−05	1.8304E−09	2.7616E−06
Median	1.6130E−04	1.6962E−04	1.6925E−04	2.4017E−04	1.4246E−03
STD	7.5164E−07	2.1953E−05	4.0141E−03	4.2783E−05	1.6618E−03

**Table 9:** Statistical indices of applied optimizers at 1.5ar/353.15 K (30 times).

Metric	ARO	GWO	PSO	SCA	SSA
Best	7.9759E−04	7.9766E−04	7.9759E−04	8.0087E−04	7.9760E−04
Worst	8.0297E−04	1.4516E−03	1.5301E−03	1.6593E−03	1.4017E−03
Average	7.9838E−04	9.0613E−04	9.1102E−04	1.0014E−03	9.3715E−04
Variance	1.5300E−12	4.1832E−08	6.4375E−08	9.1183E−08	3.0434E−08
Median	7.9783E−04	8.2446E−04	7.9762E−04	8.4111E−04	8.4151E−04
STD	1.2369E−06	2.0453E−04	2.5372E−04	3.0197E−04	1.7445E−04

**Table 10:** Results of 30 Runs at 1.0 bar/353.15 K with different optimization algorithms.

	ARO	GWO	PSO	SCA	SSA
1	2.6919E−04	2.7397E−04	7.4808E−04	3.2884E−04	3.4865E−04
2	2.6918E−04	2.7048E−04	7.4808E−04	3.4278E−04	2.8028E−04
3	2.6918E−04	2.7106E−04	2.6918E−04	4.0353E−04	2.8096E−04
4	2.6919E−04	2.7890E−04	2.6918E−04	3.0286E−04	1.4383E−03
5	2.6920E−04	2.7062E−04	7.4808E−04	3.9389E−04	4.4960E−04
6	2.6918E−04	2.7432E−04	8.0710E−04	2.7033E−04	2.9220E−04
7	2.6920E−04	2.7847E−04	7.4808E−04	3.6790E−04	3.1376E−04
8	2.6923E−04	2.8063E−04	2.6918E−04	3.1081E−04	2.8879E−04
9	2.6918E−04	2.6945E−04	7.4809E−04	4.0430E−04	3.1314E−04
10	2.6919E−04	2.7476E−04	7.4809E−04	3.6913E−04	3.8130E−04
11	2.6918E−04	2.7034E−04	2.6918E−04	3.1416E−04	3.1211E−04
12	2.6919E−04	2.6923E−04	8.0710E−04	3.3377E−04	4.7564E−04
13	2.6919E−04	2.6929E−04	7.4809E−04	3.2931E−04	2.9365E−04
14	2.6919E−04	2.8010E−04	7.4808E−04	3.1969E−04	3.7017E−04
15	2.6919E−04	2.6935E−04	1.0937E−03	4.2206E−04	5.0033E−04
16	2.6918E−04	2.6930E−04	7.4808E−04	3.7857E−04	4.0345E−04
17	2.6918E−04	2.7197E−04	7.4808E−04	3.1336E−04	4.2227E−04
18	2.6925E−04	2.7630E−04	7.4809E−04	3.1273E−04	8.7923E−04
19	2.6921E−04	2.6983E−04	8.0711E−04	5.6017E−04	4.0837E−04
20	2.6919E−04	2.6993E−04	8.0711E−04	3.2203E−04	4.8147E−04
21	2.6919E−04	2.7078E−04	9.6598E−02	7.2226E−04	4.3596E−04
22	2.6919E−04	2.6924E−04	2.6918E−04	4.5201E−04	4.7216E−04
23	2.6918E−04	2.7208E−04	7.4808E−04	3.8684E−04	2.7996E−04
24	2.6922E−04	2.6932E−04	7.4810E−04	2.8264E−04	2.6923E−04
25	2.6918E−04	2.7227E−04	7.4808E−04	2.9588E−04	2.8190E−04
26	2.6919E−04	2.6922E−04	2.6918E−04	3.8295E−04	2.8240E−04
27	2.6918E−04	2.6923E−04	7.4808E−04	2.9176E−04	4.5816E−04
28	2.6918E−04	2.7397E−04	2.6918E−04	3.3431E−04	2.8073E−04
29	2.6918E−04	2.6933E−04	2.6918E−04	3.3930E−04	2.7377E−04
30	2.6918E−04	2.7017E−04	2.6918E−04	4.2895E−04	7.6231E−04

**Table 11:** Results of 30 Runs at 1.25 bar/353.15 K with different optimization algorithms.

	ARO	GWO	PSO	SCA	SSA
1	1.6077E−04	1.7048E−04	9.6875E−03	2.8158E−04	1.4034E−03
2	1.6095E−04	1.7118E−04	2.3509E−04	2.1571E−04	2.2634E−04
3	1.6129E−04	1.8117E−04	1.6077E−04	3.1797E−04	1.0223E−03
4	1.6174E−04	1.6796E−04	9.3717E−03	2.0640E−04	1.8410E−04
5	1.6403E−04	2.0040E−04	1.6477E−04	1.9557E−04	2.2974E−03
6	1.6178E−04	2.3694E−04	1.6077E−04	2.4489E−04	3.3173E−03
7	1.6275E−04	1.6846E−04	1.7390E−04	1.7905E−04	1.1418E−03
8	1.6131E−04	1.6359E−04	1.6925E−04	2.1488E−04	2.5017E−03
9	1.6170E−04	1.6701E−04	1.6111E−04	1.8546E−04	1.4458E−03
10	1.6100E−04	1.6393E−04	1.6077E−04	2.0077E−04	1.8091E−03
11	1.6091E−04	1.6714E−04	1.6925E−04	2.8996E−04	2.0535E−03
12	1.6251E−04	1.6759E−04	1.6077E−04	1.8205E−04	1.9911E−04
13	1.6081E−04	1.7901E−04	9.3717E−03	2.5201E−04	3.3446E−03
14	1.6247E−04	1.6566E−04	2.4429E−04	2.3822E−04	2.7030E−04
15	1.6088E−04	2.1951E−04	1.6077E−04	2.6444E−04	2.7895E−03
16	1.6217E−04	1.6548E−04	1.6077E−04	2.4211E−04	6.0742E−04
17	1.6186E−04	1.7607E−04	9.3717E−03	2.9868E−04	2.5946E−04
18	1.6085E−04	1.7356E−04	1.6077E−04	2.6011E−04	3.3285E−03
19	1.6082E−04	1.6737E−04	1.6925E−04	1.7481E−04	3.8028E−03
20	1.6137E−04	2.3435E−04	9.6875E−03	2.6721E−04	2.3161E−04
21	1.6086E−04	1.6615E−04	2.4429E−04	2.7637E−04	1.8028E−04
22	1.6082E−04	1.6876E−04	1.7144E−04	3.0055E−04	5.4407E−03
23	1.6078E−04	1.7733E−04	9.3717E−03	2.1746E−04	3.2367E−04
24	1.6127E−04	2.2155E−04	1.6077E−04	2.0790E−04	1.8781E−03
25	1.6141E−04	1.6477E−04	9.6875E−03	1.8526E−04	6.7729E−03
26	1.6105E−04	1.6389E−04	1.6925E−04	1.8548E−04	1.7464E−04
27	1.6139E−04	1.6802E−04	1.6077E−04	2.6986E−04	2.1216E−03
28	1.6218E−04	1.7253E−04	1.6477E−04	2.9728E−04	2.3221E−03
29	1.6094E−04	1.7289E−04	2.3911E−04	2.1615E−04	3.4027E−04
30	1.6167E−04	2.1022E−04	1.6925E−04	2.5465E−04	4.3529E−04

**Table 12:** Results of 30 Runs at 1.5 bar/353.15 K with different optimization algorithms.

	ARO	GWO	PSO	SCA	SSA
1	7.9761E−04	7.9842E−04	1.4526E−03	8.2289E−04	1.4017E−03
2	7.9847E−04	8.0490E−04	7.9760E−04	1.5098E−03	1.1549E−03
3	7.9770E−04	8.3388E−04	7.9759E−04	8.1085E−04	8.0610E−04
4	7.9771E−04	8.2144E−04	7.9762E−04	1.6593E−03	8.0453E−04
5	7.9823E−04	8.1915E−04	8.0476E−04	1.0686E−03	8.5972E−04
6	7.9770E−04	8.5789E−04	7.9762E−04	8.9403E−04	8.0462E−04
7	7.9787E−04	8.3183E−04	7.9759E−04	8.1131E−04	9.2035E−04
8	7.9766E−04	7.9807E−04	1.5301E−03	8.0724E−04	9.6084E−04
9	7.9776E−04	8.1819E−04	1.4525E−03	1.4275E−03	8.0369E−04
10	7.9801E−04	8.2651E−04	7.9762E−04	8.1050E−04	1.0801E−03
11	7.9764E−04	8.2925E−04	7.9759E−04	8.5025E−04	8.0367E−04
12	7.9804E−04	8.2931E−04	8.0478E−04	1.5736E−03	8.0465E−04
13	7.9779E−04	1.4516E−03	7.9759E−04	8.5871E−04	1.1575E−03
14	7.9945E−04	7.9766E−04	7.9759E−04	8.4811E−04	9.8261E−04
15	7.9770E−04	8.6642E−04	8.0478E−04	1.5147E−03	8.4125E−04
16	7.9759E−04	7.9828E−04	8.0476E−04	8.7470E−04	1.2599E−03
17	7.9768E−04	8.1619E−04	1.4525E−03	1.4731E−03	8.0256E−04
18	7.9761E−04	8.3098E−04	7.9759E−04	8.1287E−04	1.0869E−03
19	7.9782E−04	8.2240E−04	8.0478E−04	8.0792E−04	7.9760E−04
20	7.9834E−04	8.0898E−04	7.9759E−04	8.0087E−04	8.0475E−04
21	7.9851E−04	8.5166E−04	7.9759E−04	8.5393E−04	1.2127E−03
22	8.0086E−04	1.4173E−03	8.0476E−04	8.0522E−04	8.0460E−04
23	7.9946E−04	1.4512E−03	7.9759E−04	8.1357E−04	8.0476E−04
24	7.9783E−04	8.3393E−04	7.9759E−04	8.0416E−04	8.0401E−04
25	8.0297E−04	1.0409E−03	1.4526E−03	8.0495E−04	9.3895E−04
26	8.0102E−04	1.2935E−03	7.9759E−04	8.2510E−04	8.4178E−04
27	7.9814E−04	8.0241E−04	7.9762E−04	1.5402E−03	7.9799E−04
28	7.9775E−04	8.0076E−04	7.9759E−04	8.0483E−04	9.3864E−04
29	7.9783E−04	8.2148E−04	8.0478E−04	8.3412E−04	1.2104E−03
30	7.9863E−04	8.0926E−04	7.9759E−04	9.2011E−04	8.2259E−04

**Fig. 6:**
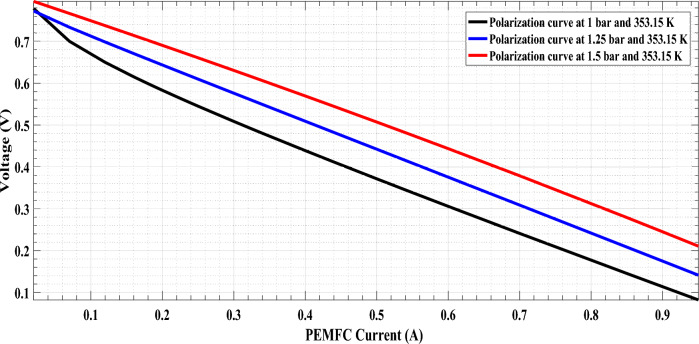
Estimated polarization (V-I) curve of PEMFC at different operating conditions.

**Fig. 7:**
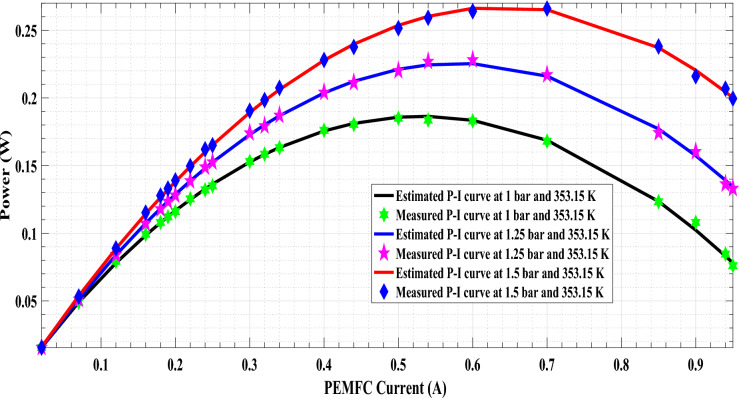
Power (P-I) curve of PEMFC at different operating conditions.

The comparison between the measured and estimated (V-I) and (P-I) curves for the 5 W PEMFC at three operating conditions (1.0 bar/353.15 K,1.25 bar/353.15 K, and 1.5 bar/353.15 K) are illustrated in Fig. 1a,b,c and Fig. 6. The best agreement between the measured and estimated curves indicates the efficiency of both the suggested model and the optimization algorithm in estimating an accurate model of PEMFC. The power of the estimated PEMFC model and the measured one is illustrated in Fig. 7. The results again show the proposed ARO algorithm’s effectiveness and accuracy in optimizing an accurate model for the PEMFC.

## Assessment study

### Sensitivity analysis

**Fig. 8:**
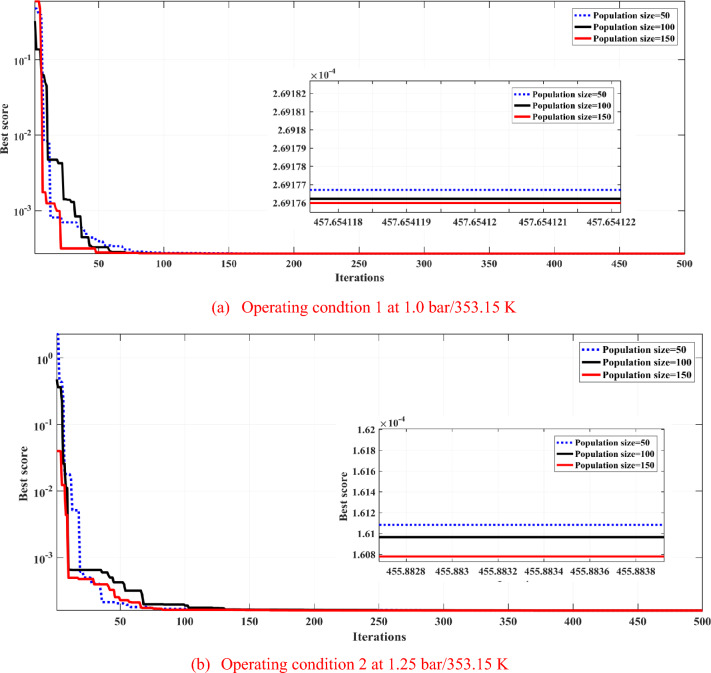
Sensitivity analysis at two operating conditions with different population sizes.

The convergence of the proposed ARO algorithm is sensitive only to the population size and the maximum number of iterations (Iter). To explain the convergence characteristics and its sensitivity to the variation in population size (P) and the maximum number of iterations (Iter), 30 independent runs are applied at each variation. Figure 8a,b explain the variation of the objective function (SSE) at two operating conditions (1.0 bar/353.15 K and 1.25 bar/353.15 K) when the population size varies as 50, 100, and 150 while the number of iterations remains at 500.

### Statistical indices

The sensitivity of ARO to a maximum number of iterations is reported in Table 13 at the considered operating conditions while the population size remains constant at 150.

**Table 13:** Statistical indices of ARO at different number of iterations (30 times).

Metric	The 1^st^ operating condition (1 bar)				The 2^nd^ operating condition (1.25 bar)			
Iteration	Iter = 100	Iter = 200	Iter = 350	Iter = 500	Iter = 100	Iter = 200	Iter = 350	Iter = 500
Best	2.699E−04	2.694E−04	2.692E−04	2.692E−04	1.649E−04	1.618E−04	1.614E−04	1.608E−04
Worst	3.028E−04	2.782E−04	2.706E−04	2.693E−04	2.567E−04	1.684E−04	1.679E−04	1.640E−04
Average	2.785E−04	2.710E−04	2.694E−04	2.692E−04	1.741E−04	1.656E−04	1.633E−04	1.615E−04
Variance	4.437E−11	2.926E−12	1.014E−13	3.143E−16	2.738E−10	3.276E−12	1.877E−12	5.650E−13
Median	2.771E−04	2.707E−04	2.693E−04	2.692E−04	1.700E−04	1.655E−04	1.631E−04	1.613E−04
STD	6.661E−06	1.710E−06	3.184E−07	1.773E−08	1.655E−05	1.810E−06	1.370E−06	7.516E−07

**Fig. 9:**
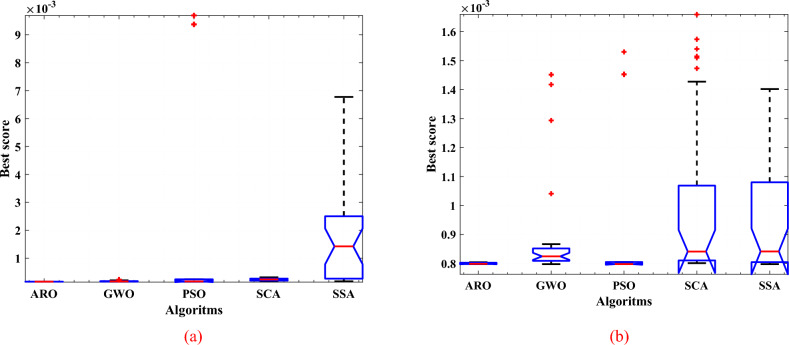
Box plot for the competitive algorithms and the proposed ARO; at (**a**) 1.25 bar/ 353.15 K, (**b**) 1.5 bar/353.15 K.

Friedman Nonparametric two-way analysis of variance and it is a non-parametric version of two-way ANOVA. The test compares the independent columns of data and returns the p value for the chi-square statistic, which explain realize the null hypothesis. Table 14 illustrates the lower values of probability (p) 2.05E−14 and 1.85E−10 at two operating conditions, 1.25 bar/ 353.15 K, and 1.5 bar/353.15 K (30 runs), respectively which are less than the null hypothesis (p < 0.05 ). In this table, the second shows the Sum of Squares (SS) due to each source. The third shows the degrees of freedom (df) associated with each source. The fourth shows the Mean Squares (MS), which is the ratio SS/df. The fifth shows Friedman's chi-square statistic. The sixth shows the p value for the chi-square statistic. Figure 9 illustrates the box plot for the competitive algorithms and the proposed ARO at two operating conditions, 1.25 bar/ 353.15 K, and 1.5 bar/353.15 K (30 runs). It is noticed that the lower mean is via ARO (the red line). Moreover, ARO has the lower variation for the SSE over 30 independent runs, compared to other algorithms.

**Table 14:** Friedman ANOVA results for different optimizers and the proposed ARO.

Conditions	Source	SS	df	MS	'Chi-sq'	'Prob > Chi-sq'
1.25ar/353.15 K	Columns	175.533	4	43.883	70.2133	2.05E−14
	Error	124.467	116	1.073		
	Total	300	149			
1.5ar/353.15 K	Columns	128.47	4	32.12	51.39	1.85E−10
	Error	171.53	116	1.48		
	Total	300	149

## Conclusion

In this work, the artificial rabbits’ optimization algorithm is proposed for finding the parameters of PEMFC. The ARO performance is verified using experimental results obtained from conducting laboratory tests on the fuel cell test system (SCRIBNER 850e, LLC). The simulation results are assessed with four competitive algorithms: Grey Wolf Optimization Algorithm, Particle Swarm Optimizer, Salp Swarm Algorithm, and Sine Cosine Algorithm. The proposed ARO algorithm has no control parameters except the setting of population size and the maximum number of iterations.The comparison between the measured and estimated (V-I) and (P-I) curves for the 5 W PEMFC at three operating conditions (1.0 bar/353.15 K,1.25 bar/353.15 K, and 1.5 bar/353.15 K) are carried out. The optimal parameters of the PEMFC are based on the electrochemical model estimated by four competitive optimization algorithms besides the lower and upper limits of the PEMFC control variables. The simulation results are reported at three operating conditions (1.0, 1.25, and 1.5 bar at 353.15 K). The extracted parameters agree with the measured ones at a lower SSE value. It is found that the ARO algorithm outperforms the others as it has the lower value of SSE (2.6918E−04, 1.6077E−04, and 7.9759E−04) at operating conditions of 1.0, 1.25, and 1.5 bar at 353.15 K; respectively. The best agreement between the measured and estimated curves indicates the efficiency of both the suggested model and the optimization algorithm in estimating an accurate model of PEMFC. The power of the estimated PEMFC model and the measured one is illustrated. Besides, the simulation results assure the superiority of ARO compared to the competitive optimizers. The convergence rate of the proposed ARO is compared to the other competitive algorithms. The proposed ARO has a fast convergence rate and the best objective function (lower SSE) compared to the other optimizers. Finally, the results again show the proposed ARO algorithm’s effectiveness and accuracy in optimizing an accurate model for the PEMFC.

## Data availability

All data generated or analyzed during this study are included in this published article.
